# Investigation of environmental ethics, spiritual health, and its relationship with environmental protection behaviors in nursing students

**DOI:** 10.17533/udea.iee.v42n1e14

**Published:** 2024-04-29

**Authors:** Mohammad Saeed Jadgal, Abdulwahid Bamri, Mojtaba Fattahi Ardakani, Nasir Jadgal, Moradali Zareipour

**Affiliations:** 1 Tropical and Communicable Diseases Research Center, Iranshahr University of Medical Sciences, Iranshahr, Iran. Email: jadgal15@gmail.com Tropical and Communicable Diseases Research Center Iranshahr University of Medical Sciences Iranshahr Iran jadgal15@gmail.com; 2 Department of public health, School of nursing, Iranshahr university of medical sciences, Chabahar, Iran Department of public health School of nursing Iranshahr university of medical sciences Chabahar Iran; 3 Assistant professor, Department of Islamic Studies, University of Sistan and Baluchistan, Zahedan, Iran. Email: avahedbameri@yahoo.com University of Sistan and Baluchestan Department of Islamic Studies University of Sistan and Baluchistan Zahedan Iran avahedbameri@yahoo.com; 4 Diabetes research center, Shahid Sadoughi University of Medical Sciences, Yazd, Iran. Email: mjfattahi57@gmail.com Shahid Sadoughi University of Medical Sciences Diabetes research center Shahid Sadoughi University of Medical Sciences Yazd Iran mjfattahi57@gmail.com; 5 Department of Nursing, School of Nursing, Iranshahr University of Medical Sciences, Chabahar, Iran. Email: nasirems7@gmail.com. Iranshahr University of Medical Sciences Department of Nursing School of Nursing Iranshahr University of Medical Sciences Chabahar Iran nasirems7@gmail.com; 6 Department of Public Health, Khoy University of Medical Sciences, Khoy, Iran. Email: z.morad@yahoo.com Corresponding author. Khoy University of Medical Sciences Department of Public Health Khoy University of Medical Sciences Khoy Iran z.morad@yahoo.com

**Keywords:** attitude, behavior, environmental ethics, environmental protection, knowledge, nursing students, spiritual health., actitud, conducta, environmental ethics, environmental protection, conocimiento, estudiantes de enfermería, spiritual health., atitude, comportamento, environmental ethics, environmental protection, conhecimento, estudantes de enfermagem, spiritual health.

## Abstract

**Objective.:**

To investigate the relationship between environmental ethics, spiritual health, and environmental behavior among nursing students.

**Methods.:**

In this cross-sectional study, 200 iranian students from the Chabahar Nursing School were selected using a simple random sampling method. The data collection tool included a questionnaire on demographic information, knowledge, attitudes and behaviors towards the environment, environmental ethics, and spiritual health. Partial least squares structural equation modeling (PLS-SEM) was utilized to evaluate the conceptual framework in this study.

**Results.:**

The mean score for environmental ethics among nursing students was 65.73±10.61 out of 100. Most of the students (47%) had desirable environmental ethics. The knowledge structure (β=0.46) predicted attitude. The attitude structure also predicted environmental behavior (β=0.28) and spiritual health (β=0.31). Ultimately, the results showed that spiritual health and environmental ethics predict environmental behavior directly and indirectly (*p*<0.001).

**Conclusion.:**

Spiritual health and environmental ethics were strong predictors of environmental behavior. Therefore, it is necessary to take into account not only students' spiritual health but also their ethical behaviors to promote environmental protection behaviors in the future.

## Resumen

**Objetivo.:**

Investigar la relación entre la ética ambiental, la salud espiritual y el comportamiento ambiental entre los estudiantes de enfermería.

**Métodos.:**

En este estudio transversal se seleccionaron 200 estudiantes iraníes de la Escuela de Enfermería de Chabahar mediante un método de muestreo aleatorio simple. La herramienta de recogida de datos incluía un cuestionario sobre información demográfica, conocimientos, actitudes y comportamientos hacia el medio ambiente, ética medioambiental y salud espiritual. Se utilizó el modelo de ecuaciones estructurales por mínimos cuadrados parciales (PLS-SEM) para evaluar el marco conceptual de este estudio.

**Resultados.:**

La puntuación media en ética medioambiental entre los estudiantes de enfermería fue de 65.73±10.61 sobre 100. El 47% de los estudiantes tenían una ética medioambiental deseable. La estructura de conocimientos (0.46) predijo la actitud. La estructura de la actitud también predijo el comportamiento medioambiental (0.28) y la salud espiritual (0.31). En última instancia, los resultados mostraron que la salud espiritual y la ética medioambiental predicen el comportamiento medioambiental directa e indirectamente (*p*<0.001).

**Conclusión.:**

La salud espiritual y la ética medioambiental fueron fuertes predictores del comportamiento medioambiental. Por lo tanto, es necesario tener en cuenta no sólo la salud espiritual de los estudiantes, sino también sus comportamientos éticos para promover conductas de protección del medio ambiente en el futuro.

## Resumo

**Objetivo.:**

Investigar a relação entre ética ambiental, saúde espiritual e comportamento ambiental entre estudantes de enfermagem.

**Métodos.:**

Neste estudo transversal, 200 estudantes da Escola de Enfermagem de Chabahar (Irã) foram selecionados usando um método de amostragem aleatória simples. A ferramenta de coleta de dados incluiu um questionário sobre informações demográficas, conhecimento, atitudes e comportamentos em relação ao meio ambiente, ética ambiental e saúde espiritual. A modelagem de equações estruturais por mínimos quadrados parciais (PLS-SEM) foi usada para avaliar a estrutura conceitual deste estudo.

**Resultados.:**

A pontuação média sobre ética ambiental entre os estudantes de enfermagem foi de 65.73±10.61 de um total de 100. 47% dos estudantes tinham uma ética ambiental desejável. A estrutura de conhecimento (0.46) previu a atitude. A estrutura da atitude também previu o comportamento ambiental (0.28) e a saúde espiritual (0.31). Por fim, os resultados mostraram que a saúde espiritual e a ética ambiental previram o comportamento ambiental direta e indiretamente *(p<0*.001).

**Conclusão.:**

A saúde espiritual e a ética ambiental foram fortes preditores do comportamento ambiental. Portanto, é necessário considerar não apenas a saúde espiritual dos alunos, mas também seus comportamentos éticos, a fim de promover comportamentos de proteção ambiental no futuro.

## Introduction

Today, human destructive activities are more threatening to biodiversity, stability, and balance of the environment than any other factor. Environmental protection has become a major challenge in developing and third-world countries.^1^ The relationship between humans and nature has never been as precarious and threatening as today. The rapid pace of technological development and changes in human lifestyles on the one hand, and the delay in economic, cultural, and ethical planning aimed at reducing their adverse effects on the other, have led to a series of environmental abnormalities and subsequent concerns among environmentalists, social thinkers and policymakers.^2^ Human environmental behaviors have caused various destructive behaviors, including the unrestricted use of energy in homes, personal transportation, single-use production, and improper waste disposal. Currently, human environmental behavior is recognized as one of the most influential factors in the environment, which has drawn the attention of many environmental sociologists.^3^^,^^4^ Environmental improvement can only be achieved when humans’ natural and cultural environments are interconnected. The necessary step towards achieving this goal is to have environmental ethics encompassing all society segments. Environmental ethics involves ideal human behavior toward their living environment, including natural, social, and cultural environments.^5^ Some researchers explicitly state that the current environmental crises are a crisis of values and ethics, which calls for an ethical solution.^5^ It is important to note that the relationship between individuals of any society with nature and the environment can be either responsible and ethical, utterly irresponsible and unethical, or sometimes something in between.^5^

Identifying the effective factors is the first step toward a change in environmental protection behavior. One of the predictors of environmental behaviors is spiritual health.^6^ Some researchers believe that spiritual health, which is the core of human health, contributes significantly to humans’ growth and development.^7^^-^^9^ Spiritual health is defined as a feeling of connection with others, having meaning and purpose in life, and having belief and connection with a higher power.^10^ Through the connection with a higher power and the creation of goals in life, spiritual health can promote responsible environmental behavior. When people feel that their behavior is under the supervision of a higher power that has created the world for the benefit of all human, plant, and animal generations, they gain a complete understanding of nature and its preservation. Accordingly, they commit themselves to protecting the environment as a top priority.^11^

On the other hand, environmental protection can be considered as one of the crucial responsibilities of students. Creating a healthy environment requires a group of students capable of building relationships with local communities and helping protect the environment. They must be enthusiastic about educating those who are indifferent to the environment or engage in environmentally risky behaviors. In short, thanks to their role as efficient actors in the social arena, students can be the founders of knowledge-raising movements, positive social movements, and appropriate environmental behavior in the field of environmental protection in society. Ministry of Health and Medical Education strongly emphasizes the spiritual health of medical students from various fields.^12^ Furthermore, spiritual health is closely associated with the development of responsible attitudes toward the environment, which ultimately affects environmental behavior.^6^ In this light, this study sought to determine the relationship between environmental ethics and spiritual health, and environmental behavior among nursing students.

## Methods

This descriptive-analytical cross-sectional study included nursing students in Chabahar as its statistical population. Also this study was conducted from October 2022 to March 2022. The inclusion criteria were as follows: willingness to participate in the study and admission to the university before September 2022. Final-year students were excluded from the study. 

According to the prevalence of 6% of the environmental protection behavior in the study of Majdi Yazdi et al.^13^ and considering the error rate of 0.05%, alpha of 5%, the sample size was determined to be 90 people. However, 200 additional participants were included in the study to compensate for any potential sample loss.




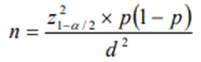




After obtaining a list of students who entered the program prior to September 2022 from the school’s education department, a random sampling of students was conducted. If some individuals were dissatisfied, the selection procedure continued randomly until the predetermined sample size (200 individuals) was reached. This study employed a demographic information questionnaire, a researcher-made questionnaire for assessing environmental knowledge and behavior, Abedi Servestani’s Environmental Ethics Questionnaire,^14^ and Paloutzian and Ellison’s standardized Spiritual Well-being Scale.^15^ The demographic questions included information related to age, gender, father’s education, mother’s education, marital status, father’s occupation, mother’s occupation, household income, and place of residence.

Section 1. Participants were asked to complete a questionnaire to assess their knowledge, attitudes, and behaviors regarding environmental issues. The questionnaire was researcher-made and consisted of 15 knowledge questions that were scored based on correct, incorrect, and “I don’t know” responses (2 points for correct, 1 point for “I don’t know,” and 0 points for incorrect responses). The total score ranged from 0 to 30. The attitude section contained 16 Likert scale questions with five response options ranging from “strongly agree” to “strongly disagree.” The questions were scored from 1 to 5, and the total score ranged from 16 to 80. A total of 14 items were included in the behavior section, which was scored based on “always,” “sometimes,” and “never” responses. The total score ranged from 14 to 42.

As a first step, the validity of the questionnaire was assessed by sending it to 10 relevant experts. They calculated the content validity ratio and index, and necessary corrections were made based on their feedback. The content validity ratio for knowledge, attitude, and behavior questions was 0.71, 0.85, and 0.83, respectively. The content validity index for knowledge, attitude, and behavior questions was 0.79, 0.71, and 0.86, respectively. Cronbach’s alpha test was used to determine the reliability of the questionnaire. The results showed Cronbach’s alpha values of 0.90 for knowledge, 0.93 for attitude, and 0.70 for behavior, which were all confirmed.

Section 2. Servestani *et al.*^14^ Environmental Ethics Questionnaire was administered, consisting of 20 questions on a five-point Likert scale ranging from “strongly agree” to “strongly disagree.” The total score ranged from 20 to 100, with scores of 20-44 indicating undesirable environmental ethics, 45-74 indicating relatively undesirable environmental ethics, and 75-100 indicating desirable environmental ethics. Regarding reliability, this questionnaire had a Cronbach’s alpha value of 0.87.

Section 3. Polotzien and Ellison’s Spiritual Well-Being Scale (SWBS)^15^ composed of 20 questions scored on a five-point Likert scale ranging from “strongly agree” to “strongly disagree.” The total score ranged from 20 to 100, with scores of 20-40 indicating poor spiritual health, 41-99 indicating average spiritual health, and 100-120 indicating good spiritual health. According to Cronbach’s alpha test, the questionnaire exhibited a scientific reliability of 0.80. SPSS 16.0 software was used to analyze the data in this study. The analysis included descriptive statistics (mean, standard deviation, frequency, and percentage) and analytical methods, such as the Kolmogorov-Smirnov test (to determine the normality of data), Pearson correlation coefficient, Spearman correlation coefficient, and multiple linear regression. Partial least squares structural equation modeling (PLS-SEM) was utilized to evaluate the conceptual framework in this study. The structural measurement model was tested using SmartPLS version 3 statistical software. Results were considered statistically significant at a level of *p*<0.05.

Ethical Considerations. Ethical approval was obtained from the Human Research Ethics Committee at the Iranshahr university of medical sciences. All study participants provided written informed consent. Confidentiality and anonymity were ensured. All procedures performed in studies involving human participants were in accordance with the ethical standards of the institutional and/or national research committee and with the 1964 Helsinki declaration. Also, this study was approved by the Research Ethics committee of Iranshahr university of medical sciences (code: IR.IRSHUMS.REC.1401.027).

## Results

A total of 130 students participated in the study, with a mean age of 21.3±1.7 years, and 62% were female. Regarding educational background, 24% of the fathers were high school graduates, and 33% were university graduates. Also, 23% of the mothers were high school graduates. An overview of demographic variables is presented in Table 1.

**Table 1:** Demographic information and baseline characteristics of study participants

Variable	Variable categories	***n* (%)**
Gender	Male	76 (38)
	Female	124 (62)
Marital status	Single	187 (93.5)
	Married	13 (6.5)
Father's education level	Illiterate	29 (14.5)
	Elementary	32 (16)
	Guidance	36 (18)
	High school	48 (24)
	University	66 (33)
Mother's education level	Illiterate	41 (20.5)
	Elementary	32 (16)
	Guidance	36 (18)
	High school	47 (23.5)
	University	44 (22)
Father's job	Employee	84 (42)
	Free	116 (58)
Mother's job	Employee	40 (20)
	Housewife	160 (80)
Income	< 2 million tomans*	18 (9)
	2 to 4 million tomans	68 (34)
	> 4 million tomans	114 (57)
Place of residence	Native	127 (63.5)
	non-native	73 (36.5)

*1Toman = 52 US dollar in 2024

The results of the current study showed that the mean score for environmental ethics among students was 65.73±10.61 out of 100. Most students (47%) had desirable environmental ethics in the classification of environmental ethics. Based on the results, the mean score for spiritual health was 94.81±13.48 out of 120. Most students (53%) had average spiritual health (Table 2).

**Table 2:** Status of Environmental Ethics and Spiritual Health among Study Participants

Range of scores	Mean ±SD	***n* (%)**	Variable categories	Variable
20-44	65.73±10.61	34 (17)	Undesirable	Environmental ethics
45-74		72 (36)	Relatively undesirable	
75-100		94 (47)	Desirable	
20- 40	94.81±13.48	7 (3.5)	Weak	Spiritual Health
41-99		106 (53)	Average	
100-120	87 (43.5)	Good

Table 3 demonstrates the relationship between demographic variables and environmental structure. Clearly, a significant positive correlation was found between age and knowledge (r=0.17, *p*<0.001). Environmental ethics was strongly correlated with attitude (r=0.48, *p*<0.001), spiritual health (r=0.61, *p*<0.001), and environmental behavior (r=0.58, *p*<0.001). Additionally, knowledge showed a significant positive correlation with environmental ethics, indicating that an increase in knowledge was associated with an increase in environmental ethics (r=0.14, *p*<0.001).

**Table 3:** Distribution of correlation between demographic variables and environmental-related constructs

	Environmental ethics	Attitude	Spiritual health	Behavior	Knowledge
Age	0.07	-0.04	0.028	-0.09	0.171*
Father's education level	-0.06	0.09	-0.07	0.14	-0.01
Mother's education level	-0.04	-0.023	0.06	0.04	0.023
Income	0.125	-0.021	-0.05	-0.03	0.14
Environmental ethics	1.00	0.48**	0.614**	0.588**	0.143*
Attitude		1.00	0.266**	0.477**	0.511**
Behavior				1.00	0.177*

(*) Weak positive correlation, (**) Strong positive correlation

**Figure 1:**
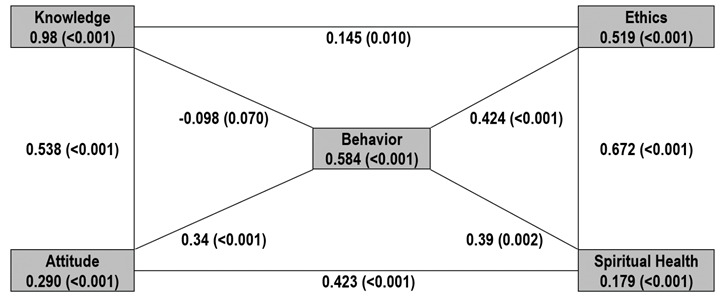
Partial least square SEM results; estimated path coefficients and their significance levels loaded on the pathways

Table 4 provides the results of the significance test for the path coefficient of the structural model. As observed, the t-values were greater than 1.64, indicating that the hypotheses are confirmed at the 90%, 95%, and 99% confidence levels. Knowledge (β=0.46) predicted environmental ethics (*p*<0.001), and environmental ethics (β=0.14) correlated with environmental behavior (*p*<0.001). Furthermore, attitude (β=0.28) and spiritual health (β=0.31) predicted environmental behavior (*p*<0.001), and spiritual health predicted environmental ethics (B=0.28, *p*<0.001). Also, environmental ethics predicted environmental behavior (β=0.42, *p*<0.001). Figure 1 illustrates the path coefficients and their significance between variables that predict environmental behavior.

**Table 4:** Results of relevant statistics for the path coefficient

Variables	Path coefficient	t values	Standard deviation (STD)	***p*-value**	Results
The relationship between knowledge and attitude	0.464	9.49	0.057	<0.001	Supports communication
The relationship between attitude and behavior	0.288	5.85	0.058	<0.001	Supports communication
The Relationship between Ethics and spiritual health	0.70	16.06	0.042	<0.001	Supports communication
The relationship between ethics and behavior	0.423	6.745	0.064	<0.001	Supports communication
The relationship between attitude and spiritual health	0.313	6.06	0.064	<0.001	Supports communication
The relationship between spiritual health and behavior	0.39	3.09	0.058	0.002	Supports communication
The relationship between knowledge and ethics	0.145	2.57	0.056	0.01	Supports communication
The relationship between knowledge and behavior	-0.098	1.82	0.054	0.07	It does not support communication

## Discussion

In the 21^st^ century, many environmental researchers have recognized human environmental behaviors as one of the most important and influential factors in the environment. The present study was conducted to determine the relationship between environmental ethics, spiritual health, and environmental behavior. The results showed that most students (47%) had desirable environmental ethics. This finding is consistent with that of Shahvali *et al*.,^16^^,^^17^ who evaluated environmental ethics in students as acceptable and desirable. Environmental ethics are determining principles that govern human-nature relationships, create internal barriers against improper actions, and convince individuals that other creatures deserve life, freedom, and enjoyment of existence.^18^ Therefore, the desirability of environmental ethics in students can effectively promote nature-appropriate conduct. 

Based on the analysis of spiritual health, most students were found to have average spiritual health (53%). Similarly, Zareipour and Gholamnia *et al*.^19^^,^^20^ reported average spiritual health among medical science students. During patient care and hospitalization, medical science students should accompany patients. Therefore, students with high spiritual health can positively influence the general health of their patients by offering support and addressing their spiritual needs, along with improving their spiritual health.

This study demonstrated that age and environmental knowledge were positively correlated. This finding is in agreement with those of Casaló *et al.*^21^ and Mirfardi *et al.,*^22^ suggesting that older individuals have a greater knowledge of their environment. Older students’ environmental knowledge, rationality, and foresight are significantly higher than younger students. Moreover, older students have more experience and knowledge about the environment, which contributes significantly to promoting responsible environmental behavior. This research examined the relationship between spiritual health, environmental ethics, and behavior. The results showed that the higher the level of spiritual health among students, the more desirable their environmental ethics and behavior were. Moreover, the hypothesis regarding the relationship between spiritual health, environmental ethics, and behavior was confirmed. Agbim *et al*.^23^ demonstrated a significant correlation between the behavioral characteristics of spiritually-minded people and their ethical behavior. Anser *et al*.^24^ also found that higher spiritual levels were associated with better environmental behavior. Kazemzadeh *et al*.^25^ reported a direct and significant statistical relationship between spiritual health and ethical behavior in students, which is consistent with the present study’s findings. Therefore, spiritual health leads to the formation of responsible attitudes towards the environment and ultimately affects the responsible environmental behavior of students.

In the present study, knowledge directly predicted attitudes, ethics, and environmental behavior. This finding is consistent with Liu and Mahboobi *et al*.’s studies,^16^^,^^26^ which demonstrated that environmental knowledge positively affects ethical and environmental behavior. Individuals with greater environmental knowledge are more sensitive to the environment. Thus, they are more likely to adopt positive attitudes and behaviors that contribute to the preservation of the environment. There is a direct and indirect relationship between attitude, environmental ethics, and environmental behavior, which is consistent with the studies by Jekria and Hansman.^27^^,^^28^ Attitude refers to individuals’ emotions, tendencies, beliefs, and judgments about environmental phenomena or events in life and their readiness to engage in environmental behavior. As the most important determinant of behavior, it is a powerful motivator for participating in environmental development activities and protecting the environment. In other words, individuals with an inclination towards environmental conservation (environmental attitudes) are more sensitive to environmental concerns. Moreover, individuals who have a positive attitude toward environmental issues are more likely to adopt environmental protection behaviors. 

Environmental ethics determines a set of principles and standards that govern human relationships with nature and aim to prevent harm to nature and protect it. These principles create internal moral deterrents that seek to correct human misbehavior towards nature, resulting in the emergence of responsible environmental behaviors in individuals. Therefore, environmental ethics lead to the promotion of responsible environmental behaviors in individuals. One of the strengths of the present study was that no study has been conducted on the relationship between environmental ethics, spiritual health and environmental protection behaviors in Iran.

Limitations. The use of self-report questionnaires as a tool for collecting information can be seen as weak points in the present study, which should be cautious in generalizing the data. Another limitation of the present study was the lack of honest cooperation of the participants. To overcome this limitation, the study participants will be reminded that the questionnaire information will be completely confidential and the results will be presented in general. According to the results of the present study, it is suggested to carry out interventional studies to ensure, maintain and improve the mental health of students with an emphasis on environmental issues.

Conclusion. The findings of the present study suggest that spiritual health and environmental ethics are strong predictors of environmental behavior. In order to promote environmental protection behaviors in the future, it is necessary to focus not only on students’ spiritual health but also on their ethical behavior and to employ solutions that enhance their ethical and spiritual behavior. It is possible to achieve desirable spiritual health and ethical behavior among students by considering the impact of these two variables on environmental behavior.
